# Coffee intake and development of pain during computer work

**DOI:** 10.1186/1756-0500-5-480

**Published:** 2012-09-03

**Authors:** Vegard Strøm, Cecilie Røe, Stein Knardahl

**Affiliations:** 1 National Institute of Occupational Health, PO box 8149, Oslo, Dep N-0033, Norway; 2 Department of Physical Medicine and Rehabilitation, Oslo University Hospital, Ullevål, Nydalen, PO box 4956, Oslo, N-0424, Norway; 3 Faculty of Medicine, The University of Oslo, Blindern, PO box 1171, Oslo, N-0318, Norway; 4 Department of Research, Sunnaas Rehabilitation Hospital, Nesoddtangen, 1450, Norway

**Keywords:** Computer work, Muscle, Pain, Coffee

## Abstract

**Background:**

The present study sought to determine if subjects who had consumed coffee before performing a simulated computer office-work task found to provoke pain in the neck and shoulders and forearms and wrists exhibited different time course in the pain development than the subjects who had abstained from coffee intake.

**Findings:**

Forty eight subjects all working fulltime, 22 with chronic shoulder and neck pain and 26 healthy pain-free subjects, were recruited to perform a computer-based office-work task for 90 min. Nineteen (40%) of the subjects had consumed coffee (1/2 -1 cup) on average 1 h 18 min before start. Pain intensity in the shoulders and neck and forearms and wrists was rated on a visual analogue scale every 15 min throughout the work task.

During the work task the coffee consumers exhibited significantly lower pain increase than those who abstained from coffee.

**Conclusions:**

Subjects who had consumed coffee before starting a pain provoking office work task exhibited attenuated pain development compared with the subjects who had abstained from coffee intake. These results might have potentially interesting implications of a pain-modulating effect of caffeine in an everyday setting. However, studies with a double blind placebo controlled randomized design are needed.

## Findings

### Background

Shoulder and neck pain occur commonly during work involving very low levels of muscle activity, such as office work with computers, with prevalence rates around 10 % [1-3]. Recently, we have reported that computer office-work performed continuously for 90 min, with time pressure and high precision demands, induced substantial pain in the shoulders and neck as well as in the forearm operating the computer mouse both in subjects with chronic pain and in healthy references [4,5]. Of the participants in this study, nearly half of them had ingested coffee before attaining the laboratory experiment. Reduced muscle pain after caffeine administration have been reported during dynamic exercise of >60% of maximal capacity [6,7] and during static grip to exhaustion tasks [8]. The present study sought to determine if subjects who had consumed coffee before starting the computer office-work exhibited different time course in the pain development than subjects who had abstained from coffee intake.

### Materials and methods

#### Subjects

Forty-eight subjects, 22 with chronic shoulder and neck pain (pain group) and 26 healthy pain-free subjects (reference group), all working fulltime, were recruited through advertisements in local papers and the Internet (for characteristics, see [4,5]).

All participants received written information and signed an informed consent. The Norwegian Regional Committee for Medical Research Ethics and the Norwegian Social Science Data Services approved the study.

#### Experimental protocol

The present study is part of a larger study investigating mechanisms of pain development during office work, however not the effect of caffeine. Extended details of the experimental protocol and procedures are presented elsewhere [4,5]. In short, the subjects had to report to the laboratory between 8 and 9 a.m. at the experimental day. To avoid decreased vigor and alertness, sleepiness, and fatigue, as have been reported when missing the regular morning coffee, the participants had the opportunity to ingest coffee or tea in conjunction with a light breakfast before attaining the laboratory. If they chose to consume coffee, they were instructed to drink no more than one cup. They were also instructed to avoid taking any kind of medication, using alcohol, and performing exhaustive physical activity the 24 hours before the experiment. When reporting to the laboratory it was registered if they had consumed coffee (yes/no), how much, and at what time. It was however not registered if they were a habitual coffee user or not.

The experimental protocol consisted of a computer-based office-work task including correcting typographical errors in a standardized text using a word processor as fast and accurately as possible for 90 min without pauses followed by 30 min recovery [4,5]. The participants were allowed to use the computer mouse *only*.

Measurements included ratings of current pain intensity in the right and left shoulder and neck and forearms and wrists on a visual analogue scale (VAS). The VAS comprised a 100 mm line anchored by the labels “no pain”; 0, and “unbearable pain”; 100 (in Norwegian “uutholdelig smerte”, a phrase commonly used in the Scandinavian language). After baseline measurements VAS-ratings were rated every 15 min throughout the work task and recovery. For extended details on the procedures and measurements, see Strøm et al. [4,5].

#### Statistics

Statistical analyses were performed using the Statistical Package for the Social Sciences (release 18.0, SPSS Inc., Chicago, IL, USA). Independent sample *t* test was used to explore between-group differences in anthropometrics. A two-tailed significance level of 5% was adopted.

To fit the best model for the time course of the dependent variable pain for the coffee consumers and coffee abstainers linear mixed model analysis was used [9]. The time was treated in the model as a continuous factor. The models were fitted either with an unstructured or a heterogeneous first-order autoregressive covariance structure based on Akaike’s information criterion for goodness of fit [9]. Separate analyses were performed to check for the influence of group (pain and reference groups).

### Results

Nineteen (40 %) of the 48 subjects reported that they had consumed coffee before attaining the laboratory (8 subjects in the pain group and 11 subjects in the reference group). The volume of coffee ingested ranged from ½ a cup to 1 cup (median 1). The mean time from coffee consumption until the experimental start was 1 h 18 min ± 28 min.

No statistically significant differences were found between the coffee consumers and the coffee abstainers regarding age, bodyweight and –height.

**Figure 1:**
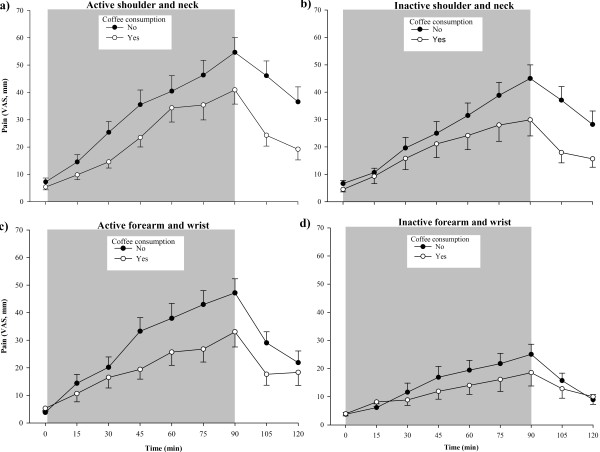
**Pain intensity.** Mean values (± SEM) of pain intensity in (**a**) the active side of the shoulder and neck, (**b**) the inactive shoulder and neck, (**c**) the active forearm and wrist, and (**d**) the inactive forearm and wrist, rated on a visual analogue scale (VAS, mm) at baseline (0) and every 15 min throughout the 90 min office work task and 30 min recovery, in subjects that had consumed coffee before start (n = 19; open circles), and in subjects that had abstained from coffee consumption (n = 29; filled circles). The shaded area indicates the 90 min work task period.

Both the coffee consumers and abstainers exhibited pain in the shoulders and neck and forearms/wrists during the work task (Figure 1). The pain increase in the active side of the shoulder/neck was significantly lower for the coffee consumers than for the abstainers (see Table 1; β5), and at the work task end the mean pain intensity was 41 ± 22 mm and 55 ± 29 mm for the consumers and non-consumer, respectively. Similar results for the pain increase were found for the inactive shoulder and neck, and the forearms/wrists. The differences in time course between the coffee consumers and abstainers were still statistically significant (*p*<0.05), when including the presence of chronic pain or not (i.e. pain and reference groups).

**Table 1:** Time course of pain intensity

	**Estimate of the β**^**b**^	**95% CI**		***p*-value**
(β1) Intercept	7.5	5.2	9.8	< 0.001
(β2) Coffee_i_^c^	−3.5	−7.2	0.2	0.064
(β3) Time_ij_^d^	7.3	5.9	8.7	< 0.001
(β4) BP90_ij_	−16.6	−21.1	−12.1	< 0.001
(β5) Coffee_i_ * Time_ij_	−2.9	−5.1	−0.7	0.011
(β6) Coffee_i_ * BP90_ij_	3.4	−3.8	10.6	0.34

Estimated regression coefficients with 95% confidence intervals (CI) based on the fitted linear models^a^ of the pain intensity ratings (VAS, mm) in the active side of the shoulder and neck during and after performing the 90 min computer work in subjects that had consumed (n = 19) or abstained (n = 29) from coffee before start.

^a^Linear mixed models for repeated measurements fitted with unstructured covariance structure.

^b^β2: the difference between coffee consumers and abstainers at baseline; β3: the change per 15 min time period during the 90 min work task; β4: BP90 = break point 90 min, i.e. the change from the work task ending and through each 15 min period during the 30 min recovery; β5: the difference between coffee consumers and abstainers for each 15 min period during the work task; β6: the difference between coffee consumers and abstainers per 15 min periods during the recovery. ^c^0 = No; 1 = Yes. ^d^0 = Baseline; 1 = 15 min; 2 = 30 min, and so on.

### Discussion

The present results revealed significant lower increase in the pain development during the computer office-work task for the subjects who had consumed coffee approximately 1 ½ h before the task than subjects that had abstained from coffee.

The present study was originally undertaken to investigate mechanisms of pain development during computer office work [4,5], and not the effect of coffee on pain. Thus, the present results must be viewed within the limitations of the study regarding the effect of the coffee consumption. Since a controlled randomized design of the coffee consumption was not used a lot of uncertainties about the association between coffee intake and differences in pain-perception during the office work task exist. The exact dose of coffee is unknown; the quantity of coffee consumed was self-reported even though one cup was the maximal allowed limit; the size of a cup may vary and also the caffeine dose [10], and blood samples were not taken to measure the caffeine in the system. The time from coffee intake to experimental start was not standardized. The mean time spent from coffee consumption until start was, however, within the half-life of caffeine [10]. Nevertheless, the attenuated pain response seen for the coffee drinkers was statistically significant for all measured pain sites even when adjusted for the presence of chronic pain, and a difference in VAS pain score >14 mm can be considered as clinically significant [11]. Furthermore, the size of the effect of caffeine on pain attenuation in the active shoulder/neck indicates a moderate effect (Cohen’s d = 0.54). Thus, we hypothesize that the coffee consumption may have had an attenuating effect on the pain development during the computer work.

The reason for allowing the coffee intake before starting the experiment was to avoid unpleasant effects of caffeine deprivation, e.g. decreased vigor and alertness, sleepiness, and fatigue, as have been reported when missing the regular morning coffee in habitual coffee drinkers [12,13]. It is reasonable to believe that those who ingested coffee before start were habitual coffee drinkers. However, it is not known if those who not ingested coffee also were habitual coffee users or not.

We are not aware of studies that have examined the effect of coffee consumption on naturally occurring pain during work of very low-level muscle activity, as during computer work. However, several studies have reported attenuated muscle pain after caffeine administration during dynamic exercise of >60% of maximal capacity [6,7], and during ischemic [14] and eccentric muscle contractions [15]. The caffeine doses in these studies were >5 mg/kg, and probably higher than what is presumed doses in the present study.

A single cup of coffee provides a caffeine dose of 0.4 to 2.5 mg/kg [10] and most of the caffeine is absorbed from the gastrointestinal tract within 45 min. For doses lower than 10 mg/kg the half-life of caffeine is between 2.5 and 4.5 h [10]. Caffeine is metabolized in the liver to form the methylxanthines paraxanthine and theophylline [16]. Caffeine is an adenosine receptor antagonist [10]. Adenosine receptors are distributed throughout the nervous system, in the vascular endothelium, heart, liver, adipose tissue, and in the skeletal muscle [17-19].

The potential mechanism of caffeine on the pain response in the presents study is uncertain. Caffeine has high affinity to both A_1_ and A_2A_ receptors [10]. Adenosine may exert its influence on nociception via peripheral C-fiber involvement and within the central nervous system [20]. Activation of adenosine A_1_ receptors inhibits nociception both in the periphery and at the spinal level, while activation of A_2A_ receptors augments nociception peripherally, but seems to have no direct effect on nociception at the spinal level [20]. The action of caffeine thus depends on the type of receptors that is blocked and on tissue in which the receptors are located.

In conclusion, subjects who had consumed one cup of coffee before starting a pain provoking office work task exhibited attenuated pain development compared with the subjects who had abstained from coffee intake. These results might have potentially interesting implications of a pain-modulating effect of caffeine in an everyday setting. However, studies with a double blind placebo controlled randomized design are needed.

## Competing interests

The authors declare that they have no competing interests.

## Authors’ contributions

All authors participated in the design and conception of the study. VS and CR conducted the laboratory work. VS analyzed the data and drafted the manuscript. All authors read, revised and approved the final manuscript.
